# Supplementary material for: A high-resolution mRNA expression time course of embryonic development in zebrafish
Source: eLife. 2017 Nov 16;6:e30860. doi: 10.7554/eLife.30860 (PMC5690287; doi:10.7554/eLife.30860)
Supplement: Supplementary file 6. [file elife-30860-supp6.zip › biolayout-clusters-files/Cluster150-genes.html]

Cluster150


# Cluster150: Genes

| Ensembl ID | Gene Name | Chr | Start | End | Biotype |
| --- | --- | --- | --- | --- | --- |
| ENSDARG00000076557 | bicc2 | 14 | 2690680 | 2728761 | protein\_coding |
| ENSDARG00000029179 | foxd1 | 5 | 34397610 | 34399400 | protein\_coding |
| ENSDARG00000079293 | gdpd4b | 21 | 22275130 | 22288542 | protein\_coding |
| ENSDARG00000003165 | nr2f6b | 11 | 6118366 | 6136421 | protein\_coding |
| ENSDARG00000074253 | olig3 | 13 | 1175060 | 1177729 | processed\_transcript |
| ENSDARG00000042032 | pou3f3a | 9 | 6680609 | 6683681 | protein\_coding |
| ENSDARG00000009387 | robo4 | 10 | 31358279 | 31394209 | protein\_coding |
| ENSDARG00000075467 | shisa3 | 14 | 46212992 | 46219704 | protein\_coding |
| ENSDARG00000096639 | si:ch211-101l18.8 | 20 | 19643679 | 19645969 | processed\_transcript |
